# The potential of immature jackfruit in meat analogues

**DOI:** 10.1016/j.crfs.2025.101255

**Published:** 2025-11-24

**Authors:** Anne C.M. Swinkels, Maarten A.I. Schutyser, Atze Jan van der Goot

**Affiliations:** a Fiber Foods, Johan Huizingalaan 763A, Amsterdam, 1066 VH, the Netherlands; b Laboratory of Food Process Engineering, Wageningen University and Research, P.O. Box 17, Wageningen, 6700 AA, the Netherlands

**Keywords:** Jackfruit (*Artocarpus heterophyllus*), Immature, Meat analogue, Plant-based, Hybrid, Structure, Fibre

## Abstract

The **jackfruit**, the fruit of the **jackfruit** tree (***Artocarpus heterophyllus***), is a unique tropical fruit. While sweet and fruity in its ripe form, in its **immature** form, the **jackfruit** flesh mimics the texture of meat, making it an increasingly popular **plant-based meat analogue**. To reach its full potential as an ingredient for **meat analogues**, a better understanding of the **immature** fruit properties in relation to its behaviour in food products is required.

This review focuses on **immature jackfruit** as an ingredient in meat-like applications. Specifically, we discuss the processing of **immature jackfruit**, its use in **plant-based meat analogues** and **hybrid** meat products, the impact of its implementation on textural and sensorial attributes, and the nutritional composition of the fruit. We conclude with an outlook on challenges and opportunities for research and applications that can boost the implementation of **jackfruit** as an ingredient in **plant-based** foods.

Traditionally, **immature jackfruit** has been used as a vegetable in various cuisines. Current preservation methods include the addition of preservatives and the application of heat treatment. **Immature jackfruit** has a unique fibrous structure that closely resembles meat and has been successfully incorporated into **plant-based meat analogues** and **hybrid** products. While its low protein content may limit its role as a standalone **meat analogue**, the dietary **fibre** content of **jackfruit** offers valuable nutritional benefits that support its inclusion in a more balanced, **plant-based** diet.

## Highlights

• Immature jackfruit has a unique fibrous structure similar to meat.
• Preservation strategies for immature jackfruit include antioxidant treatments and thermal processing.
• Immature jackfruit in plant-based and hybrid foods show promising results in product quality and consumer acceptance.
• Further research is needed to improve processing and expand immature jackfruits' use in food applications.

## Graphical abstract

**Figure:**
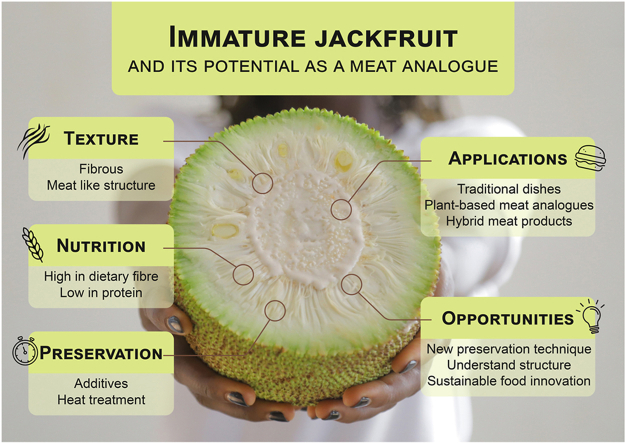

## 1 Introduction

**Fig. 1:**
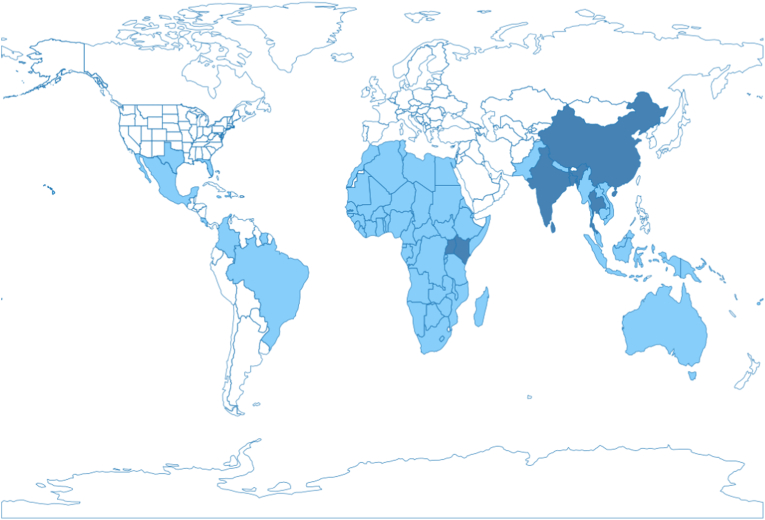
World map with countries growing jackfruit in light blue and leading countries in dark blue.

The jackfruit tree, or jak tree, botanically referred to as *Artocarpus heterophyllus*, is a tropical tree that belongs to the Moraceae family. It is a large, tropical evergreen tree that can grow up to 20 m tall (Khan et al., 2021; Kuldeep et al., 2023) and produces the world's largest tree-borne fruit, the jackfruit (Ranasinghe et al., 2019). Jackfruit trees grow in tropical regions worldwide (Fig. 1) and require a warm, humid climate. The tree is native to the Western Ghats of India and grows abundantly in India, Sri Lanka, Bangladesh, Thailand, and regions of China (Haq and Hughes, 2002; Verheij and Coronel, 1991). It is also commonly found in Uganda and Kenya (Morton, 1987) and to a lesser extent in other African and Asian countries, as well as in parts of the Americas and Australia (Orwa et al., 2009; Mondal et al., 2024; Madruga et al., 2014; Barros-Castillo et al., 2021; Kaur et al., 2024; Elevitch and Manner, 2006; Palipane and Rolle, 2008; Top Countries for Jackfruit Export).

**Fig. 2:**
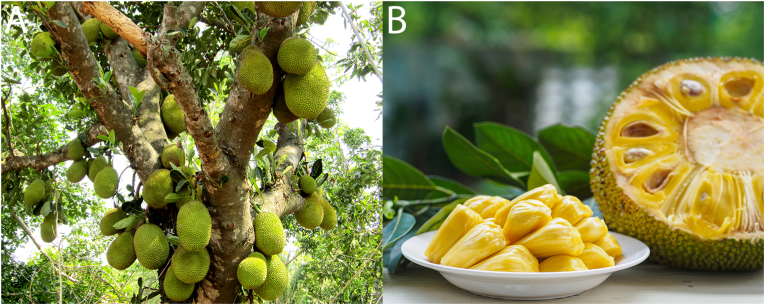
A: Jackfruit tree. B: Mature jackfruit.

Jackfruits grow directly on the trunk of the tree and vary significantly in size (Hossain et al., 2012). Their length ranges from 30 to 100 cm, their diameter is between 15 and 50 cm, and their weight can reach up to 55 kg (Ranasinghe et al., 2019; Morton, 1987; Elevitch and Manner, 2006). The exterior of the jackfruit is green and consists of hexagonal, conical carpel apices that form a thick peel (Fig. 2A). The jackfruit is a climacteric fruit, ripening both on and off the vine (Ranasinghe et al., 2019). In addition, it is a multiple fruit, meaning that the fruit is formed from the fusion of multiple flowers. The fruit's interior, therefore, consists of numerous seeds that are covered by yellow carpels (Fig. 2B). When ripe, the carpels have a soft texture and a sweet and fruity aroma (Grimm and Steinhaus, 2019).

The edible bulbs of the jackfruit only make up 40 % of its weight (Subburama et al., 1992). As a result, a large portion of the fruit is wasted, making valorisation of jackfruit by-products an important research area, although the large-scale implementation of those ideas remains limited (Kalse and Swami, 2022), (Pragalyaashree et al., 2022). Currently, the peel of the jackfruit is locally used as compost and has applications in the chemical industry (Tran et al., 2021; Kalse and Swami, 2022). The seeds, commonly consumed after boiling or roasting (Chai et al., 2021), are suitable for the industrial extraction of valuable protein and oil fractions for use in cosmetic, chemical, or food applications (Brahma and Ray, 2023; Waghmare et al., 2019). Moreover, the white stripes of ripe jackfruit that sit between the fruit bulbs have potential as an ingredient in meat analogues (Hamid et al., 2020). Also, jackfruit flour, made by drying and milling the rind of jackfruit, has applications as a binder ingredient in meat analogues, bread and cookies (Kaila, 2023; Felli et al., 2018; Ramya et al., 2020). Lastly, the most common application of jackfruit and its by-products is as feed for cattle, rabbits, and fish (Kusmartono, 2007; Orwa et al., 2009; Effiong et al., 2024; Roslan and Mesa, 2023). Beyond fruit production, the jackfruit tree serves other purposes, such as preventing soil erosion, acting as a windbreak, providing shade, and offering timber for ornamental purposes (Elevitch and Manner, 2006). The leaves of the tree have potential as a source for proteins and phytochemical extracts (Indrianingsih et al., 2024; Moreno-Nájera et al., 2020). Thus, the applications of jackfruit by-products are diverse; however, large-scale implementation remains limited. By-product applications would bring additional commercial value to the fruit. It's estimated that only 30 %–50 % of the jackfruits produced are used for food applications (Pragalyaashree et al., 2022; Kaur et al., 2024; Kalse and Swami, 2022). This appears to be due to the high vulnerability and degradation of the fruit, combined with a lack of awareness, inadequate post-harvest technology, and complications in the supply chain, such as difficulties in maintaining controlled temperature conditions and poor handling (Ranasinghe et al., 2019; Sundarraj and Ranganathan, 2018).

**Fig. 3:**
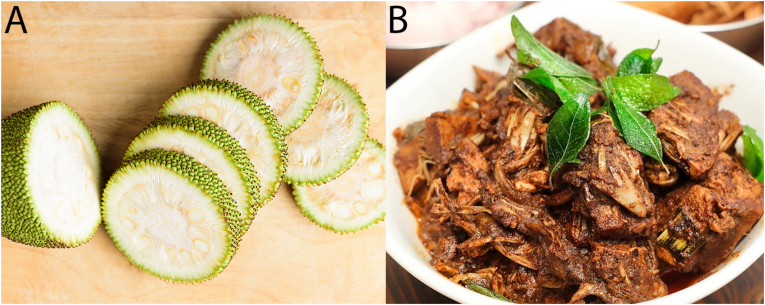
A: Immature jackfruit B: Jackfruit dish mimicking the structure of pulled pork from Rathnayake (2020).

In recent years, utilising jackfruit has emerged as a promising strategy in the transition to a more plant-based diet and improved food security. Immature jackfruit, also referred to as unripe, young, or baby jackfruit, has a unique fibrous structure that highly resembles the structure of meat (Fig. 3) (Ong et al., 2021); therefore, the interest in the fruit as a plant-based meat analogue is growing. Unfortunately, there is a lack of standardisation of the harvest maturity index for immature jackfruit (Kaur et al., 2024), though several indicators exist to determine whether a fruit is still immature or is in the next stage of ripening. Ripe fruits can be recognised by a change of colour from green to green-yellowish, a hollow sound when tapped by the finger, and a change in outer skin spikes. Generally, the rule of thumb is that fruit matures in about 12–16 weeks after flowering (Palipane and Rolle, 2008; Ramli, 2009). To describe and compare jackfruit ripeness, researchers classified jackfruit into different maturity stages. Sometimes this classification is “immature” and “mature”, whereas others classify the jackfruit into 6 stages (Sidhu, 2012). Often, as well as in this review, the classification from Sidhu is used, and we assume stage 1 (no fruitlets or seeds are formed) and stage 2 (fruitlets and seeds are just starting to develop) jackfruit to be immature jackfruit (Sidhu, 2012). Riper fruits are not considered in this review because the focus is on the use of jackfruit in meat-like applications.

The objective of this review is to discuss the current status, challenges and opportunities for the processing and use of immature jackfruit in meat analogues and hybrid meat applications. We will map the usage of immature jackfruit, including its traditional usage and current processing methods. Additionally, we will discuss its more recent applications as a structural ingredient in meat analogues and hybrid meat products, highlighting the effects on textural and sensorial properties. Furthermore, the nutritional composition of the jackfruit is discussed, and we identify challenges and highlight opportunities for future research and food processing with immature jackfruit.

## 2 Traditional usage and processing

Traditionally, immature jackfruit is consumed as a cooked vegetable, in soups or in (coconut) curries in several Asian cuisines (Saxena et al., 2011; Rathnayake, 2020). Additionally, it is commonly pickled in brine or vinegar with a mustard seed seasoning (Saxena et al., 2011). In India, immature as well as overripe and fallen fruits, along with jackfruit leaves, are fed to livestock (Orwa et al., 2009). Jackfruit is often referred to as “the poor man's fruit” or “starvation fruit”. It played an important role in the 1970s as a food source during the famines in Sri Lanka (Rathnayake, 2020). In both Sri Lanka and Nepal, immature jackfruit is more commonly consumed than the ripe variety. In fact, 60 % of jackfruit in Nepal is eaten as a green vegetable (Haq and Hughes, 2002).

Cleaning and cutting jackfruit are labour-intensive due to the sticky latex it releases when cut (Shidenur et al., 2024). Selling pre-cut and well-preserved jackfruit adds value to the fruit, extending its usability beyond local consumption. However, this approach demands effective preservation techniques, as jackfruit is highly susceptible to degradation and has a limited shelf life under typical conditions. One of the major limitations of jackfruit is its rapid browning upon cutting, caused by the enzyme polyphenol oxidase (Navindra et al., 2009).

Discolouration of immature jackfruit can be mitigated by applying anti-browning solutions. A combination of 1.5 % citric acid and 1.5 % ascorbic acid, or 3 % citric acid with 1 % sodium metabisulphite, was found to be optimal for preventing browning. The preservatives also enhanced the microbiological quality of the fruit over time, facilitating sales in local supermarkets and storage for up to seven days (Ekanayaka et al., 2015). In another study, calcium chloride at concentrations of 2 and 5 % was effective in preventing browning and extending the shelf life of the immature fruit (Rana et al., 2018a). Mishal et al. (2022) used potassium metabisulfite to avoid the browning of jackfruit at an undefined concentration. Citric acid (1 %) and ascorbic acid (2 %) reduced browning in a study by Navindra et al. (2009), extending the shelf life of the fruit if combined with polyethene pouches and cold storage (2–4 °C), up to 15 days. This minimal processing and specific storage conditions might suffice for certain situations, but further processing can extend its shelf life under more conditions.

The common preservation method for immature jackfruit is thermal processing (Mishal et al., 2022; Samutsri and Thimthuad, 2025; Samutsri et al., 2023). Traditionally, this was done through boiling in dishes; however, it now also includes pasteurisation and sterilisation during canning (Sushama Babu et al., 2022; González-Regalado et al., 2024). Sushama Babu et al. (2022) studied the effect of different thermal conditions on the canning of jackfruit. The canning was found to be microbiologically effective in preserving the fruit, but it did affect quality parameters. A longer exposure to higher temperatures negatively affected colour, texture and antioxidant activity. González-Regalado et al. (2024) studied the effect of different boiling and steaming conditions on the textural properties of jackfruit. Hardness, chewability and shear force decreased with increasing temperatures and processing times. Alongside boiling and steaming, other preservation methods such as frying, flaking, and drying have been reported, but not supported by detailed scientific studies (Swami et al., 2012; John et al., 1993; Ambiliy and Davis, 2016; Saxena et al., 2011).

## 3 Processed meat-like applications

Immature jackfruit is recognised by its meaty texture, making it an increasingly popular structural ingredient in hybrid meat products and plant-based meat analogues. However, despite widespread claims of its similarity to meat, scientific proof for this remains limited. Recent studies have begun to explore this gap: González-Regalado et al. (2024) found that immature jackfruit that is boiled (90–100 °C for 5–15 min) or steamed (121 °C for 5–10 min) has comparable textural properties to meat. Additionally, image analysis by Ong et al., 2021 showed that jackfruit fibres visually resemble those of traditional beef. Still, the structural and textural similarities between jackfruit and meat are not well-defined. Nonetheless, more research is now available on jackfruit in food applications where it is used to mimic a meat structure.

### 3.1 Plant-based meat analogues

Recent studies described the use of jackfruit in plant-based meat analogues (Table 1). Within those studies, a distinction can be made between two different types of studies: 1) studies that have investigated the effect of jackfruit addition on textural attributes of the product, and 2) studies that have investigated sensory attributes and consumer acceptance of the products. Moreover, the physicochemical characteristics of the products were tested in most studies. Additionally, a separate study focused on preserving jackfruit in a plant-based application using various drying techniques (Leelawat and Taikerd, 2024). To the best of our knowledge, this is the only article that focuses on further processing for applications with jackfruit, rather than formulating an application.

**Table 1:** Overview of previous scientific studies on plant-based and hybrid applications with jackfruit as an ingredient.

	Product type	Jackfruit percentage [%]	
**Plant-based application**	Chicken meat analogue (pea)	50, 55, 60	Leelawat and Taikerd (2023)
	Chicken meat analogue (soy)	30–50	Taikerd and Leelawat (2023)
	Chicken meat analogue (soy)	40	Leelawat and Taikerd (2024)
	Plant-based meat analogue	22, 23, 24, 25, 26, 27, 28	Mishal et al. (2022)
	Plant-based meatball	27	Samutsri and Thimthuad (2025)
	Vegan chicken sausage	0, 30, 60	Keerthana Priya et al. (2022)
	Vegan nugget	0, 25, 50, 75, 100	Xin et al. (2024)
	Vegan patty	34–38	Madhav et al. (2025)
	Vegan sausage	20, 40, 60, 80	Paranagama et al. (2022)
**Hybrid application**	Beef meat	0, 25, 50, 100	Chi et al. (2024)
	Beef sausage	0, 25, 50, 75, 100	Faujan et al. (2022)
	Chevon patties	0, 10, 20, 30	Verma et al. (2015)
	Chicken balls	0, 5, 10, 15	Samutsri et al. (2023)
	Chicken patties	0, 10, 20	Ismail-Fitry and Abas (2019)
	Embutido (beef)	0, 50	Mosura and Esbieto (2022)
	Jerky (mackerel)	35, 50, 65	Agustin and Yilistiani (2024)

**Fig. 4:**
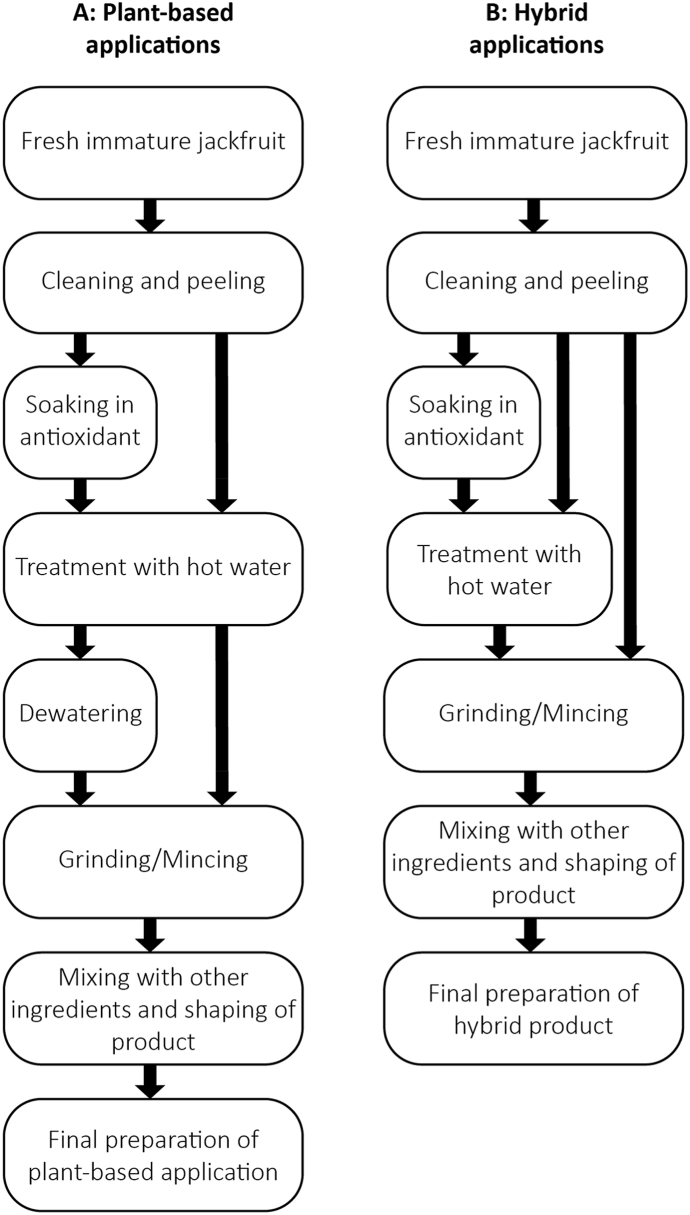
Process flow diagram for plant-based (A) and hybrid (B) applications with jackfruit in previous scientific studies.

To use jackfruit in a meat analogue, it is processed first (Fig. 4A). Jackfruit is usually first cleaned, peeled, and treated with warm water. Boiling/soaking temperatures range from 50 °C to 100 °C, with times varying from 10 to 35 min. In some instances, additional processing steps are described, such as a soaking step in metabisulfite (Mishal et al., 2022; Madhav et al., 2025) or citric acid (Samutsri and Thimthuad, 2025) before boiling. Also, dewatering through squeezing (Samutsri and Thimthuad, 2025) and a combination of pressing and mild drying after boiling are applied (Leelawat and Taikerd, 2024; Madhav et al., 2025; Leelawat and Taikerd, 2023; Taikerd and Leelawat, 2023). Depending on the specific food application, other processing steps are applied to the boiled jackfruit. It always involves grinding or blending the jackfruit to reduce its size, mixing it with other ingredients, and subsequently extruding or moulding the dough into a patty or sausage. Products are stored frozen or cooled before final preparation, which involves cooking (plant-based meatball (Samutsri and Thimthuad, 2025)), frying (plant-based chicken sausage (Keerthana Priya et al., 2022), vegan nuggets (Xin et al., 2024), vegan sausage (Paranagama et al., 2022)), steaming (chicken meat analogues from soy and pea (Taikerd and Leelawat, 2023), (Leelawat and Taikerd, 2023; Leelawat and Taikerd, 2024)) or a combination of steaming and frying (vegan patty (Madhav et al., 2025)).

Taikerd and Leelawat (2023) studied the textural attributes of different combinations of jackfruit, wheat gluten, and soy protein in a chicken meat analogue. It was observed that the microstructure of the sample with more jackfruit exhibited fibre-like characteristics and contained some pores between the fibre strands, loosening the product structure. Further, an increased content of jackfruit decreased the hardness and chewiness of the meat analogue. This was due to the high moisture content of jackfruit compared to the other ingredients, which softens the product. Hamid et al. (2020) and Leelawat and Taikerd (2023) reported similar findings in a meat analogue with jackfruit, wheat gluten and soy, and a meat analogue with hydrated pea protein isolate and jackfruit, respectively. Samutsri & Thimthuad varied the boiling time of jackfruit to study the effect on hardness, springiness and chewiness of plant-based meatballs (Samutsri and Thimthuad, 2025). They found that an increased boiling time decreased the hardness and springiness, due to heat extraction of pectin. The pectin formed gel-like structures in the meatball, which explains why the removal of pectin leads to a softer texture in the product. In the study of Keerthana Priya et al. (2022), hardness, adhesiveness, resilience, cohesion, springiness, gumminess and chewiness were also measured. In contrast to the other studies, hardness increased with increasing jackfruit content, while chewiness decreased. However, the inclusion of banana floret as an additional ingredient may explain why these trends differ from those reported in other articles.

In addition to quantifiable textural properties, sensory attributes are relevant for applications with jackfruit, shaping the overall eating experience and consumer acceptance. Keerthana Priya et al. (2022) reported lower scores compared to the control (meat sausage) in taste, flavour, chewiness and overall acceptability for the plant-based chicken sausage with 30 % and 60 % jackfruit. However, the overall acceptability and other parameters were still good. The main reason for the reduced liking was the difference in taste, as the meat analogue missed the unique juiciness and tenderness of meat products. Paranagama et al. (2022) described differences in odour, texture, taste, and overall acceptability between vegan sausages with varying jackfruit-to-mushroom ratios. For taste, texture, odour, colour and overall acceptability, the 60:40 sausage scored higher than the 20:80 sausage, suggesting that a higher amount of jackfruit is more favourable in this application. Also in the vegan patty of Madhav et al. (2025), the product was preferred at higher concentrations (up to 38 %) of jackfruit, in appearance, flavour, juiciness, chewiness, taste and overall acceptance. In contrast, Mishal et al. (2022) concluded that with 25 % and 26 % jackfruit, their application was most acceptable after studying different percentages of jackfruit in a plant-based meat analogue. Their panel described that an increase in the amount of jackfruit improved the appearance, texture, flavour, mouthfeel, aftertaste, and overall acceptability. However, above a jackfruit percentage of 26 %, the liking started to decrease due to changes in chewiness, binding, juiciness, and hardness of the product. Taikerd and Leelawat (2023) reported that increasing the jackfruit percentage did not significantly affect how fibrous the product was perceived by the participants, despite more fibre-like characteristics being seen in the microstructure. However, texture as a parameter was perceived differently among samples. Also, appearance, taste and overall acceptability were influenced by the jackfruit content. They concluded that the most favourable percentage of jackfruit was around 40 % (Taikerd and Leelawat, 2023; Leelawat and Taikerd, 2023). Lastly, Samutsri and Thimthuad (2025) saw that an increase in boiling time of jackfruit decreased the fibrous structure in the end application. The application with the least fibrous texture scored higher for appearance, colour, flavour, taste, texture and overall satisfaction. Overall, jackfruit shows potential as a structural ingredient in plant-based meat analogues, particularly due to its fibrous texture. However, its optimal inclusion level is product-specific, as excessive jackfruit can impact texture, binding, and flavour. Balancing jackfruit with the other ingredients is thus key to formulating the ideal meat analogue.

### 3.2 Hybrid meat applications

Hybrid meat applications contain a combination of meat and plant-based ingredients, with varying ratios depending on the specific application. Immature jackfruit has been implemented into hybrid meat applications as a plant-based ingredient, due to its unique structural properties, and has been studied by several researchers (Table 1). Within those studies, a distinction can be made between two different types of studies, which can occur both singly or combined: 1) studies that investigated the effect of jackfruit addition on textural attributes of the product, and 2) studies that investigated sensory attributes and consumer acceptance of the products.

To use jackfruit in a hybrid application, it is processed slightly differently in comparison with plant-based applications (Fig. 4B). However, the exact processing of jackfruit for hybrid applications is less well reported. In three articles, jackfruit is cleaned, cut, and thereafter boiled, with boiling times ranging from 2 to 40 min (Samutsri et al., 2023; Chi et al., 2024; Ismail-Fitry and Abas, 2019). After cooking, a mincing or blending step is applied to the jackfruit. In one of those articles, jackfruit is soaked in water with 0.3 % citric acid before boiling (Samutsri et al., 2023). Two articles report using chopped/minced jackfruit, without specifying if any heat treatment was applied (Verma et al., 2015; Mosura and Esbieto, 2022). The other two articles only report the use of immature jackfruit, without specifying how it was processed (Faujan et al., 2022; Agustin and Yilistiani, 2024). In all cases, jackfruit is mixed with other ingredients with a mincer, chopper, grinder, or blender for product formation. In a few articles, a cooling or freezing step is applied after the product formation. Directly after formation or after thawing, products are either boiled (chicken balls (Samutsri et al., 2023), beef meat (Chi et al., 2024)), grilled (chicken patties (Ismail-Fitry and Abas, 2019), chevon patties (Verma et al., 2015)), or heated in a steam oven or oven (beef sausage (Faujan et al., 2022), embutido (Mosura and Esbieto, 2022), jerky (Agustin and Yilistiani, 2024)).

In chicken balls, the addition of jackfruit decreased both hardness and springiness of the product (Samutsri et al., 2023), due to the lower protein content and higher moisture content of jackfruit compared to chicken meat. Similar findings were observed in chevon patties (Verma et al., 2015) and plant-based meat analogues (Taikerd and Leelawat, 2023). In contrast, Ismail-Fitry and Abas (2019) and Faujan et al. (2022) did not find differences in hardness and springiness with increasing jackfruit concentration in chicken patties and beef sausages. Although they used comparable percentages of jackfruit, this difference might result from the specific application and presence of other ingredients. Samutsri et al. (2023) observed that as the level of jackfruit increased, the meat dough became less cohesive. They hypothesised that this was due to the replacement of protein with fibre, as fibre does not bind fat as effectively. Chi et al., 2024 described that the substitution of jackfruit resulted in a more stable meat dough, which is contrary to what was reported by Samutsri et al. (2023). Here, the increased dietary fibre content was thought to improve water retention and oil absorption.

The sensory evaluation of meat products with jackfruit added yielded varying results across different studies. In the study by Samutsri et al. (2023), the taste, flavour, and overall preference of meatballs with 5 % and 10 % jackfruit did not differ significantly from the control sample without jackfruit. Conversely, Ismail-Fitry and Abas (2019) found that texture, flavour, and overall preference were improved with the addition of jackfruit at both 10 % and 20 %. Additionally, Verma et al. (2015) observed an increase in scores for appearance, odour, juiciness, texture, tenderness, flavour, and overall acceptability with increasing jackfruit percentage. Only at the highest percentage of jackfruit (30 %) did the scores start to decrease. Other studies, though, reported different outcomes. Agustin and Yilistiani (2024) noted that with an increased amount of jackfruit, the texture of restructured mackerel meat was evaluated less favourably by the panel. Faujan et al. (2022) reported that consumers preferred beef sausages without jackfruit, mainly due to decreased scores for aroma and taste. Similarly, in embutido, a Filipino-style meatloaf, Mosura and Esbieto (2022) found that the addition of jackfruit led to a lower product score. Although the texture was perceived as similar, the general acceptability shifted from “liked extremely” to “liked moderately”, primarily because the aroma and taste were described as jackfruit-like at 50 % jackfruit substitution. Despite these less favourable sensory outcomes, the authors concluded that the addition of jackfruit was generally accepted and beneficial from both cost and nutritional perspectives. These contrasting outcomes highlight the complexity and variability in the sensory perception of meat products with added jackfruit. Factors such as the specific application, percentage of jackfruit added, flavour profile, and the type of meat and other ingredients used are likely to contribute to these differences. Understanding these nuances is crucial for optimising the use of jackfruit in hybrid meat applications to meet consumer preferences.

## 4 Nutritional composition

Immature jackfruit is used as an ingredient in hybrid meat or plant-based meat analogues and it is essential to consider the impact on the nutritional value of the resulting products. They are rich in carbohydrates and fibre, low in fat and could be part of a healthy diet. There are several articles and books describing the nutritional composition of immature jackfruit. The composition of jackfruit is known to change with ripening, during which the carbohydrate content increases (Konsue et al., 2023). This change in composition makes it difficult to compare the composition of jackfruits from different studies with varying ripeness levels. Besides differences in ripeness, other factors, such as soil type, climate conditions, harvest season, and tree variety, influence its composition. Nevertheless, an approximate composition of jackfruit, referred to as stages 1 and 2 or up until 10 weeks after flowering (Table 2). An elaborate overview of articles describing the composition can be found in appendix 1 (macronutrients) and appendix 2 (micronutrients).

**Table 2:** Approximate nutritional composition of immature jackfruit.

Components	g/100 g
Moisture	85.3–89.9
Protein	0.5–1.9
Carbohydrates	5.2–10.4
Fibre	2.5–5.3
Fat	0.1–1.0
Ash	0.4–1.1

The protein content of immature jackfruit is widely reported as below 2 %, while the amino acid composition of the fruit has been measured by Konsue et al. (2023). Immature jackfruit, at stages 1 and 2, is mainly rich in aspartic acid (2169.00 and 1509.50 mg/100 g) and proline (1150.50 and 1050.56 mg/100 g). The most abundant essential amino acid is lysine (579.89 and 469.89 mg/100 g), followed by leucine (534.87 and 407.96 mg/100g). Compared to other fruits, jackfruit is relatively high in protein, but protein level is lower when comparing it to traditional meat products (Konsue et al., 2023), (Bohrer, 2017), which may necessitate supplementation or combination with another protein-rich ingredient to meet specific dietary needs. Due to its low levels of protein, iron and vitamin B12, the Dutch Nutrition Centre has classified jackfruit as an unsuitable meat analogue (Voedingscentrum). This nutritional shortfall is a concern in the shift towards a more plant-based diet, as meat analogues are usually expected to meet the dietary requirements of meat. However, protein intake in the Western world often exceeds nutritional needs (Alexander et al., 2017; Dagevos, 2021). A focus exclusively on protein hinders the development of more sustainable and diverse food products, overlooking other critical dietary needs. For example, many European consumers do not consume enough dietary fibre, leading to significant health risks (Stephen et al., 2017). Jackfruit, being high in fibre and low in protein, has the potential to address this dietary imbalance, particularly when consumed combined with meat. Therefore, the jackfruit appears to be a promising ingredient for the hybrid meat market. As discussed earlier, hybrid products containing jackfruit have been well-received for their texture and sensory attributes. Thus, incorporating jackfruit into meat products can reduce protein intake while increasing fibre consumption, without significant compromises on texture and taste.

The composition of the dietary fibre of jackfruit was researched by Rahman et al. (1999). They found that immature jackfruit contains more insoluble dietary fibre than soluble fibre, primarily due to its high cellulose content in the cell wall. The dietary fibre content and composition did not vary between the soft and firm varieties, but the fibre content decreased with ripening, as also shown in jackfruit by Konsue et al. and Rahman et al., as well as in other fruits (Konsue et al., 2023; Rahman et al., 1995; Phillips et al., 2021).

Jackfruit is also recognised for its mineral and vitamin content (Table 3). An elaborate overview of articles describing the micronutrients of immature jackfruit can be found in appendix 2. Across all the research found, the focus was often on different minerals. Calcium, ranging from 28.4 to 63.4 mg/100 g fruit, was the only component measured by all researchers. The reported values fall within a wide range, which is likely due to differences in maturity, the origin of the fruits, their specific cultivar, soil nutrients, and the season of harvesting.

**Table 3:** Approximate micronutrient content of immature jackfruit.

**Micronutrient**	**Content in mg/100 g**

Ca	28.4–63.4
Mg	37.4–37.8
Cu	0.3–2.0
Fe	0.4–4.2
Mn	0.6–0.9
Pb	0.08–0.3
Zn	2.44^a^
Na	1.5–19.4
P	2.3–35.2
K	190.6–323.0
Vitamin C	2.2–19.7
Vitamin B1	3.9–14.2
Vitamin B2	35.7–124.2

	****Content in IU****

Vitamin A	30.0–44.4

a Only mentioned in Chandra and Bharati (2020).

Jackfruit is often mentioned as a source of vitamin C, but the vitamin content differed to a large extent throughout the found studies, from 2 to 19.7 mg/100 g. The vitamin C content of jackfruit is known to increase upon ripening (Ranasinghe and Marapana, 2019). Its concentration might not be the highest in the immature fruit, but it is present in a concentration comparable to papaya. However, there are fruits with a higher vitamin C content, like citrus (Hernández et al., 2006).

Jackfruit contains a range of phytonutrients such as carotenoids, flavonoids, volatile acids, sterols, and tannins. These compounds are recognised for their antioxidant properties, which play a crucial role in protecting the body against oxidative stress and inflammation. The concentration of these phytonutrients depends on the fruit's age and variety. It was found that antioxidant activity generally correlates with total phenolic and flavonoid content. The total phenolic content is highest in stage 2 fruits, while the total flavonoid content peaks in stage 1 fruits, with both decreasing upon ripening (Konsue et al., 2023). The bioavailability of these antioxidants upon digestion was found to be good, indicating that immature jackfruit is a health-promoting ingredient with effective antioxidant properties. Polyphenols are retained after processing into vegan patties (Madhav et al., 2025); however, the impact of such processing on flavour and mouthfeel has not been thoroughly investigated.

While the nutritional composition and health benefits of jackfruit are promising, the effects of processing on these nutrients should be carefully considered. Current processing methods used in the production of plant-based meat analogues or hybrid meat products often include boiling, which results in the loss of water-soluble compounds, such as sugars and vitamins, that leach into the cooking water. Additionally, some phytonutrients and antioxidants, such as vitamin C, are heat-sensitive and might degrade during thermal processing. Hence, some of the health benefits associated with jackfruit may be diminished after boiling, although this is not discussed in recent literature. This highlights the need for a deeper understanding of how different processing techniques affect the nutritional profile of jackfruit. Moreover, there is an opportunity to explore the recovery and reuse of nutrient-rich cooking water, for example, through drying or incorporating it into other food applications, to minimise nutrient loss. Alternatively, food processing methods could be designed or optimised to retain these valuable nutrients better, ensuring that jackfruit-based products deliver both functional and nutritional benefits.

## 5 Conclusion and future outlook

Recently, immature jackfruit has been gaining more attention in literature as a potential structural ingredient. The texture of jackfruit is highly valued, especially in applications where its fibrous structure mimics meat, making it a versatile ingredient in various products. Not only dishes with chunks of jackfruit in them, such as curry, but also hybrid meat products and plant-based meat analogues benefit from jackfruit's structure. However, as a meat analogue, jackfruit doesn't have the same protein content as meat. Nevertheless, its high fibre and low fat content present a unique opportunity, particularly as dietary fibre intake becomes more emphasized in the European diet. Additionally, considering the rising prices of meat, jackfruit may be a valuable structural alternative.

A deeper understanding of the structural similarities between jackfruit and meat is needed to strengthen its role in plant-based diets. In particular, gaining more insights into the fruit's texture, fibrousness and compositional characteristics can enhance its appeal and facilitate its incorporation into a broader range of food applications. Such knowledge could also identify other fibrous crops with similar properties, collectively driving the food industry toward more sustainable and nutritious meat analogues.

There is a concern with the amount of jackfruit that goes to waste. Due to its short shelf life, preservation is necessary; however, well-established preservation methods beyond cooking are limited. Exploring alternative preservation techniques such as dehydration, freezing, fermentation, pickling, salting, or innovative packaging methods holds great potential. Strengthening and diversifying preservation methods can enhance jackfruit's contribution to food security, as it has done in traditional food systems. Advancing these methods to ensure jackfruit remains shelf-stable without compromising its colour, flavour, and texture is crucial for expanding its global distribution and increasing accessibility. Additionally, understanding how these preservation methods affect the functional properties and nutrient profile of jackfruit is key.

## CRediT authorship contribution statement

Anne C. M. Swinkels: conceptualization, writing – original draft, writing – review & editing.

Maarten A. I. Schutyser: conceptualization, writing – review & editing, supervision.

Atze Jan van der Goot: conceptualization, writing – review & editing, supervision.

## Declaration of competing interest

The authors declare the following financial interests/personal relationships which may be considered as potential competing interests: Anne Swinkels reports a relationship with Fiber Foods that includes: employment. If there are other authors, they declare that they have no known competing financial interests or personal relationships that could have appeared to influence the work reported in this paper.

## Figures and Tables

**Table 4:** Overview of recent articles describing the nutritional composition of immature jackfruit

Moisture	Protein	Fat	Ash	Fibre	Carbohydrates	Sugars	Starch	Fruit age	Country and region	Source
**[%]**	**% Dry weight basis [g/100g]**									
88.7	12.2	4.0	12.5	20.0	51.3			6 weeks after flowering	Thailand	Konsue et al. (2023)
88.4	11.7	3.6	11.3	19.3	52.9			9 weeks after flowering	Thailand	
87.7			5.2	43.4		1.2	7.8	7–8 weeks after flowering, soft variety	Bangladesh, Jessore	Rahman et al. (1999)
88.3			4.8	42.5		–	9.0	7–8 weeks after flowering, hard variety	Bangladesh, Jessore	
88.2			3.6	43.7		2.3	8.7	7–8 weeks after flowering, soft variety	Bangladesh, Dhaka	
89.3			3.8	45.0		3.3	11.1	7–8 weeks after flowering, hard variety	Bangladesh, Dhaka	
87.4			5.7	47.1		1.9	9.9	7–8 weeks after flowering, soft variety	Bangladesh, Comilla	
86.9			3.8	46.0		5.5	11.1	7–8 weeks after flowering, hard variety	Bangladesh, Comilla	
**[%]**	**[g/100 g sample]**									
89.40 ± 4.60	2.1 ± 0.43	0.14 ± 0.03		4.4 ± 0.02	12.4 ± 0.90			Stage 1, hard variety	India	Rana et al. (2018b)
92.80 ± 4.60	1.1 ± 0.56	0.92 ± 0.22		2.1 ± 0.10	19.6 ± 0.50			Stage 1, soft variety	India	
84.20 ± 3.60	2.4 ± 0.64	0.09 ± 0.07		3.1 ± 0.50	12.5 ± 0.90			Stage 2, hard variety	India	
91.10 ± 3.60	1.3 ± 0.44	1.60 ± 0.42		2.1 ± 0.10	18.3 ± 0.70			Stage 2, soft variety	India	
86.0								10–11 weeks after flowering, soft variety	Bangladesh	Rahman et al. (1995)
87.5								10–11 weeks after flowering, hard variety	Bangladesh	
70.61 ± 0.0	3.43 ± 0.08	4.16 ± 0.05	3.15 ± 0.02	9.96 ± 0.01	89.27 ± 0.02			7 weeks after flowering	India	Chandra and Bharati (2020)
68.43 ± 0.08	4.36 ± 0.06	2.43 ± 0.04	3.53 ± 0.01	7.16 ± 0.13	89.68 ± 0.02			10–12 weeks after flowering	India	
86.18 ± 0.85						3.055 ± 0.967	1.597 ± 0.295	6–7 weeks after flowering	Sri Lanka	Ranasinghe and Marapana (2019)
89.21 ± 2.29						5.125 ± 1.268	1.918 ± 0.267	8–10 weeks after flowering	Sri Lanka	
82.00 ± 0.19	1.90 ± 0.25	0.19 ± 0.08	0.83 ± 0.01	3.52 ± 0.14				Undefined	Indonesia	Agustin and Yilistiani (2024)
87.88						5.25		40 days after fruit onset	Bangladesh	Saha et al. (2016)
87.18						6.31		50 days after fruit onset	Bangladesh	
85.51						7.45		60 days after fruit onset	Bangladesh	
85.2	2	0.6	0.7	2.6	11.5			“Young”	-	Verheij and Coronel (1991)

**Table 5:** Overview of recent articles describing the micronutrients [mg/100 g sample] in immature jackfruit

Ca	Mg	Cu	Fe	Mn	Pb	Zn	Na	P	K	Fruit age	Country	Source
28.4	37.8	0.28	4.24	0.56	0.08					45 days (6.5 weeks) after flowering	-	Ranasinghe et al. (2019)
29.86	37.38	0.26	2.64	0.56	0.32					55 days (8 weeks) after flowering	-	
55.30 ± 0.28		2.00 ± 0.25	2.75 ± 0.04	0.94 ± 0.08		2.44 ± 0.04				7 weeks after flowering	India	Chandra and Bharati (2020)
54.6 ± 2.60							1.46 ± 0.43	26.1 ± 2.50	254.4 ± 2.50	Stage 1, hard variety	India	Rana et al. (2018b)
43.8 ± 1.80							12.1 ± 0.38	2.3 ± 0.60	190.6 ± 4.50	Stage 1, soft variety	India	
63.4 ± 3.00							8.43 ± 0.32	35.2 ± 1.60	226.6 ± 4.10	Stage 2, hard variety	India	
49.6 ± 1.40							19.4 ± 0.86	14.3 ± 1.80	220.8 ± 3.60	Stage 2, soft variety	India	
53			0.4				3	20	323	“Young”	-	Verheij and Coronel (1991)

**Table 6:** Overview of recent articles describing the vitamin content in immature jackfruit

Vitamin A [IU]	Vitamin C [mg/100 g]	Vitamin B1	Vitamin B2	Fruit age	Country	Source
39.4 ± 3.60	18.65 ± 0.24			Stage 1, hard variety	India	Rana et al. (2018b)
44.4 ± 2.60	12.12 ± 1.22			Stage 1, soft variety	India	
31.2 ± 4.30	16.98 ± 0.36			Stage 2, hard variety	India	
32.8 ± 1.60	9.84 ± 1.32			Stage 2, soft variety	India	
30	12.0			“Young”	-	Verheij and Coronel (1991)
	2.18 ± 0.34			Stage 1, 6–7 weeks after flowering	Sri Lanka	Ranasinghe and Marapana (2019)
	3.14 ± 0.98			Stage 2, 8–10 weeks after flowering	Sri Lanka	
	18.5	3.9	35.7	45 days (6.5 weeks) after flowering	-	Tiwari and Vidyarthi (2015)
	19.67	14.2	124.2	55 days (8 weeks) after flowering	-	
	4.48			40 days after fruit onset	Bangladesh	Saha et al. (2016)
	6.27			50 days after fruit onset	Bangladesh	
	9.30			60 days after fruit onset	Bangladesh	
